# Composites Electrodes Based on Castor Oil Derivatives and Graphite: Synthesis, Properties and Electroanalytical Applications

**DOI:** 10.1021/acsomega.5c10053

**Published:** 2025-12-10

**Authors:** Jonatha de Freitas, Rafael da Silva, Rafael Martos Buoro, Rafael Turra Alarcon, Éder Tadeu Gomes Cavalheiro

**Affiliations:** 153988 Universidade de São Paulo-USP, Instituto de Química de São Carlos, 13566-590 São Carlos, São Paulo, Brazil

## Abstract

In this study, solid composite electrodes based on an epoxidized and maleinized castor oil polymer (ECO-MECO) and graphite were prepared and characterized to evaluate their thermal, morphological, and electrochemical properties. Composites were formulated with varying graphite contents (50–85 wt %). Thermogravimetric analysis (TGA) confirmed the homogeneity of the composites as well as their graphite contents, revealing two main mass loss events corresponding to the decomposition of the agglutinant and the subsequent oxidation of graphite. Scanning Electron Microscopy (SEM) images showed a progressive increase in the amount and distribution of graphite lamella with higher graphite content, indicating improved dispersion and interfacial interaction. Contact angle measurements demonstrated that the addition of graphite reduced surface hydrophobicity, enhancing wettability. Electrochemical performance was investigated using cyclic voltammetry (CV) and electrochemical impedance spectroscopy (EIS), with results compared to a glassy carbon electrode (GCE). Composites containing 70% and 80% graphite (wt %) exhibited comparable or superior peak currents relative to GCE. The 70% graphite (wt %) composite was selected for preparation of electrodes and surface activation studies. After electrochemical treatment in phosphate solutions at different pH values, the electrode showed enhanced redox responses under both anodic and cathodic probes compared to GCE. Bode-phase analysis revealed a shift toward lower frequencies, indicating increased capacitive behavior. This transition was attributed to the formation of oxygenated functional groups on the electrode surface, which enhance charge storage and interfacial reactivity. Differential pulse voltammograms of Pb^2+^ and dopamine revealed that the analytical signal can be enhanced by preconcentration step and surface treatment, respectively. These results highlight the potential of biopolymer–graphite composites as sustainable materials for electrochemical sensing applications.

## Graphical abstract

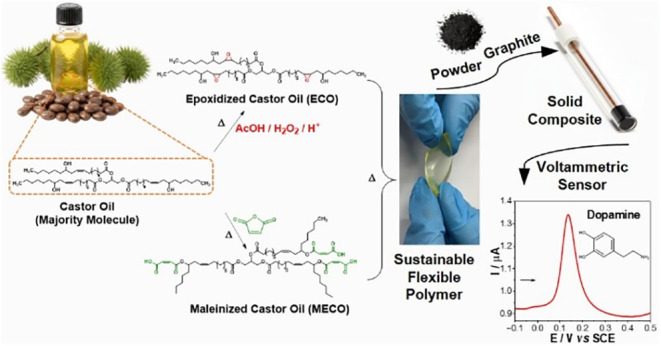

## 1 Introduction

Graphite-based composite electrodes were introduced as a viable alternative to metals such as mercury and gallium used in the past, due to the restricted potential window, adverse environmental issues and health impacts. In this context, composite electrodes offer several advantages, including ease of fabrication, surface renewability, mechanical stability and chemical inertness in nonaqueous solutions.^1–3^ They also provide low-cost fabrication, long-term durability, customizable shapes and dimensions, and the unique ability to incorporate modifiers both at the surface and throughout the bulk.^1,2^

In this regard, composite electrodes emerge as an attractive alternative, not only due to their performance advantages, but also because their composition can be prepared to reduce environmental impact. Tallman and Petersen defined composite electrodes as “*a material consisting of at least one conductor phase comingled with at least one insulator phase*”,^4^ where the insulator also serves as an agglutinant for conductive material, providing mechanical support and maintaining the structural integrity of the composite.

The conductive component in composite electrodes is most often composed of graphite and its derivatives, which rank among the most prevalent materials in conductive pastes and composites.^5,6^ Types of carbon-based materials mostly used in composite electrodes include carbon black,^7^ carbon nanotubes,^8^ fullerenes,^8^ acetylene black,^9,10^ and graphene.^11,12^ These materials are favored not only for their remarkable electrical conductivity but also for their low cost and reduced environmental impact when compared to metallic alternatives, especially rare or heavy metals.

On the other hand, a wide range of insulator/agglutinant materials have been reported in the literature for use in composites. Common examples include mineral oils such as *nujol*,^13,14^ silica or hybrid organosilicates using sol–gel matrix^15^ and various polymeric binders like epoxy resins,^16,17^ polypyrrole,^18,19^ polyesters,^20^ polydimethylsiloxane,^21^ polyacrylonitrile,^22,23^ and polyurethane,^24^ among others. Although these agglutinants offer desirable mechanical and chemical properties, the majority are derived from nonrenewable petroleum-based sources. Moreover, even when alternative or so-called “green” reagents are employed, they are often associated with toxic byproducts or components. This contradiction highlights a critical gap between current material practices and the principles of Green Chemistry, emphasizing the urgent need for sustainable and nontoxic agglutinant systems in composite electrodes.

In this context, castor oil was selected as the primary vegetable oil due to its abundance, low depletion, and rich composition in ricinoleic acid.^25,26^ Extracted from the *Ricinus communis* plant seeds, castor oil represents a promising feedstock for the development of greener methodologies in polymer synthesis and the preparation of composite electrodes,^27,28^ while it does not compete with other oils for the food chain. In this study, epoxidation and maleinization reactions were employed to increase the reactivity of castor oil, as the native oil exhibits limited functional group reactivity. Both modifications were carried out following the principles of Green Chemistry.

Despite other biopolymer-graphite composite electrodes are described in the literature,^29,30^ most of such electrodes are rigid and do not allow the preparation of flexible devices.

The reaction between epoxidized castor oil and maleinized castor oil proceeds by the nucleophilic ring opening of the epoxy group in the presence of carboxylic acid groups derived from maleic anhydride, resulting in the formation of polyesters.^31^ This approach enables the development of novel polymers, that can be used as agglutinants in composites. Notably, this strategy supports the design of material with 100% biobased content, aligning with the United Nations’ Sustainable Development Goal 12: Responsible Consumption and Production^32^

The novelty of this work is exploring the chemical modification of castor oil to enhance its reactivity and enable the synthesis of a new biobased material for use as an agglutinant in the preparation of solid composite electrodes with graphite powder. Thus, three different graphite-to-agglutinant ratios were investigated and compared to the glassy carbon electrode. Additionally, surface treatments using phosphate solutions at various pH were used to evaluate and enhance the electrochemical response of the resulting devices, and followed by the responses regarding both negatively (ferricyanide) and positively (hexaammineruthenium­(III)) charged redox probes. To address its performance as an electrode material the responses of an inorganic (Pb^2+^) and an organic probe (dopamine) on the new electrode surface were demonstrated.

## 2 Experimental Section

### 2.1 Reagents and Solutions

Amberlite IR120, hydrogen peroxide (50% H_2_O_2_), glacial acetic acid (≥99%), potassium chloride (≥99%), maleic anhydride (≥99%), graphite powder (<20 μm), potassium ferrocyanide (≥99%), potassium ferricyanide (≥99%), and hexammineruthenium­(III) chloride (≥98%), dopamine hydrochloride (≥98%) were purchased from Sigma-Aldrich and used without further purification. Castor oil was obtained from Univar (Brazil). Standard stock solution of Pb­(II) (1000 mg L^–1^) from SpecSol, Brazil.

All solutions were prepared with water deionized in an OS 10 LZ reverse osmosis device (GEHAKA, Brazil) and purified in a Barnstead system (resistivity ≥18.2 MΩ·cm) without oxygen removal.

Phosphate solutions were prepared with potassium phosphate monobasic (≥99.0%) and sodium phosphate dibasic (≥98.0%), both from Spectrum. The solutions were prepared and the pH was adjusted to the desired value using 1.0 mol L^–1^ NaOH and 1.0 mol L^–1^ H_3_PO_4_ solutions.

### 2.2 Instrumentation

Electrochemical measurements using direct current and alternating current techniques were carried out on an Autolab PGSTAT 204 potentiostat/galvanostat equipped with EIS module, connected to a microcomputer controlled by NOVA software version 2.1.3 (both from Metrohm, Switzerland), using a conventional three-electrode thermostatized glass electrochemical cell with a volume of 25.0 mL.

Scanning electron microscopy (SEM) micrographs were obtained using a LEO440 scanning electron microscope equipped with a 7060 OXFORD system (Cambridge, U.K.), operating at 15 kV. Samples were fixed onto aluminum stubs using carbon tape and coated with a 3 nm layer of gold using a MED 020 sputter coater (BAL-TEC).

Thermogravimetric analyses were performed on a Q50 instrument, controlled by the Advantage for Q-Series v.5.5.23 software (both from TA Instruments), using approximately 6.0 mg of sample (0.1 μg precision), weighed directly in the thermobalance and using a heating rate of 10 °C min^–1^ in open platinum pan. The furnace atmosphere was nitrogen (90 mL min^–1^) from room temperature up to 600 °C, thereafter the atmosphere was changed to dry air (90 mL min^–1^) and the analyses were executed up to 1000 °C.

Dynamic Mechanical Analysis (DMA) was carried out using a Q800 analyzer (TA Instruments). Polymer specimens were prepared with dimensions of 20.0 mm in length, 5.00 mm in width, and 1.00 mm in thickness, submitted to a ramp from 0.10 to 3.0 N min^–1^. Tensile test (stress–strain curve) for the polymer was performed on this DMA instrument at 25 °C.

Contact angle measurements were conducted using a C201 Attension Theta Flex optical tensiometer, controlled by the One Attension software (Biolin Scientific, Sweden), equipped with a digital camera from Navitar (Navitar). A droplet was deposited on the sample surfaces, and 50 optical scans were performed to determine the contact angle formed between the droplet and the surface.

The pH measurements were performed using a digital pH meter, model 827 pH Lab, connected to a combined glass electrode Ag|AgCl|KCl (3.0 mol L^–1^), model 6.0228.010 (both from Metrohm, Switzerland). All measurements were carried out at room temperature.

### 2.3 Esterification and Epoxidation of Castor Oil

The preparation of the polymer from maleinized (MECO) and epoxidized (ECO) castor oil is already described.^33–36^

Maleic esterification was conducted by reacting 100 g of the castor oil with 28.01 g of maleic anhydride.^37^ The reaction mixture was maintained at 100 °C for 6 h under reflux conditions in a round-bottom flask equipped with a magnetic stirring bar.^37^ Following the reaction, the mixture was transferred to a separatory funnel and washed with water and Na_2_CO_3_ (0.10 mol L^–1^) solution until a pH = 7.0 was achieved. The resulting solution was dried using MgSO_4_ and filtered to give the final maleic esterified castor oil (MECO).

Epoxidation was subsequently performed according to the methodology outlined in a previous study.^38,39^ Thus, 100 g of castor oil, 118.8 mL of hydrogen peroxide (50%), 19.98 mL g of glacial acetic acid, and 10.0 g of Amberlite IR120 catalyst were added to a round-bottom flask equipped with a magnetic stir bar. The reaction mixture was stirred for 4 h at 80 °C. After completion, the crude product was filtered to remove the catalyst and extracted with ethyl acetate. The organic layer was washed five times with Na_2_CO_3_ (0.10 mol L^–1^) and once with NaCl (0.50 mol L^–1^). Finally, the organic layer was dried using solid MgSO_4_ under low pressure to afford the final epoxidized product (ECO) as a white viscous liquid.

### 2.4 Preparation of Graphite/Polymer Composite Electrodes

In this work equimolar amounts of MECO and ECO were mixed with graphite, resulting in five graphite:polymer mass ratios: 50:50, 60:40, 70:30, 80:20, and 85:15 (% w/w) in order to prepare 1.0 g of the composites. Table 1 presents the mass of monomers and graphite used for each tested composition, along with the respective curing time and temperature conditions.

**Table 1:** Mass, Curing Temperature, and Time Used in the Preparation of Polymer and Composites with Different Compositions

GEST^a^ (%)	graphite (g)	ECO^b^ (g)	MECO^c^ (g)	*T*_cure_ ^d^ (°C)	*t*_cure_ ^e^ (min)
**0**	-	0.180	0.220	125	150
**50**	0.50	0.225	0.275		
**60**	0.60	0.180	0.220		
**70**	0.70	0.135	0.165		
**80**	0.80	0.090	0.110		
**85**	0.85	0.068	0.082		

a GEST = solid composite of graphite and esterification reaction.

b ECO = epoxidized castor oil.

c MECO = maleinized castor oil.

d *T*_cure_ = curing temperature;

e *t*_cure_ = curing time.

For the preparation of the graphite/polymer (wt %) composites, the components were thoroughly homogenized in a porcelain mortar and pestle and then transferred to a metallic mold fitted with a 3.0 mm diameter extrusion orifice. The mixture was compressed using a manual press and then extruded to produce cylindrical rods 3.0 cm in length. Such procedure was repeated three times to ensure uniform dispersion of graphite within the polymer matrix. The extruded rods were then cured in an oven at 125 °C for 2.5 h.

After curing, the rods were sectioned into 1.0 cm segments, which were subsequently named GEST composites—an acronym referencing graphite (G) and the esterification (EST) reaction between the modified oils. Electrical contact was established by attaching a copper wire (8.0 cm length, 1.0 mm diameter) to the composite with a conductive silver epoxy (Cat. No. 12,642–14, Conductive Silver Epoxy Kit, Electron Microscopy Sciences). The assembly was cured at 25 °C for 24 h using a support to maintain alignment between the wire and the composite.

Following this, the composite/wire assembly was inserted into a glass tube (5 mm inner diameter, 7 cm length), filled with epoxy resin (Silaex 6400, Brazil), and cured at 25 °C for an additional 24 h. This same procedure was applied to prepare the other compositions. Figure 1 illustrates a schematic representation of the oil modifications and composite electrode preparation.

**Figure 1:**
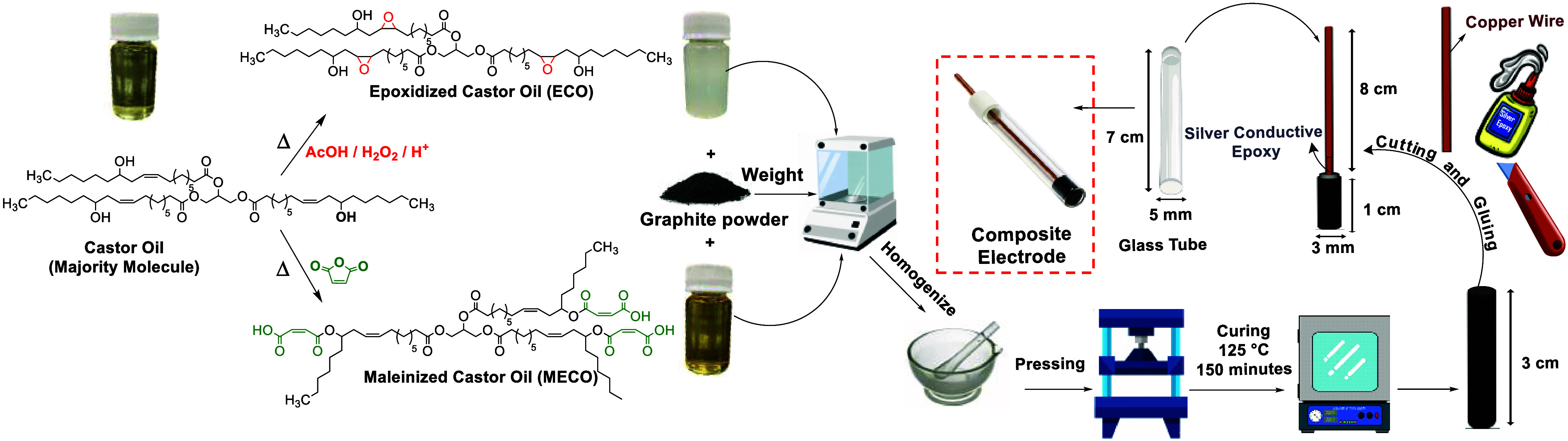
Schematic of monomer synthesis and fabrication of the solid composite electrode.

The successful composite electrodes labeled as G60EST40, G70EST30, and G80EST20 were molded to ensure a geometric surface area of 0.071 cm^2^ (3.0 mm diameter) and used as working electrodes. A platinum foil (0.55 cm^2^) and a saturated calomel electrode (SCE) were employed as counter and reference electrodes, respectively.

Before use the resulting devices were polished with 600 and then 2000 grit sandpaper in a polishing wheel (AROPOL-2 V, Arotec Brazil). After that they were sonicated in an ultrasonic bath (LISC 1400, Unique) in water for 5 min to remove residual particles.

### 2.5 Surface Electrochemical Treatment

Cyclic voltammetry was used to both characterize the composites surfaces from the proposed composite ratios and evaluate the activation procedure for the best composite among them. The activation procedure was carried out for the chosen composite using a 0.10 mol L^–1^ phosphate buffer solution at three different pH values, 3.0, 4.0, and 7.0. The activation was carried out by applying up to 300 cycles within a potential interval ranging from −1.0 to +1.5 V (vs. SCE), using a scan rate of 200 mV s^–1^. The resulting voltammograms were compared with those obtained from a mirror-like surface of a glassy carbon electrode, without any prior treatment. All electrodes used had the same physical dimensions, with equivalent geometric area and diameter.

### 2.6 Evaluation of the Electrochemical Response at the Activated Surface

The cyclic voltammetry (CV) was performed using potassium ferricyanide and hexaammineruthenium­(III) chloride solutions as electrochemical probes. For the ferricyanide-based probe, the solution was prepared by dissolving the corresponding components in a 1:1 molar ratio of K_4_[Fe­(CN)_6_]:K_3_[Fe­(CN)_6_], and resulting in final concentration of 1.0 mmol L^–1^ for the iron complexes with addition of supporting electrolyte (KCl 0.50 mol L^–1^). Measurements were performed over a potential range from −0.3 to +0.7 V (vs SCE) at a scan rate of 50 mV·s^–1^. For the hexaammineruthenium­(III) chloride probe and supporting electrolyte, the solution was prepared with 1.0 mmol L^–1^ and 0.50 mol L^–1^ (KCl), respectively. Measurements were conducted over a potential interval from −0.55 to +0.1 V, using the same scan rate of 50 mV s^–1^.

Prior to the Electrochemical Impedance Spectroscopy (EIS) analysis, the half-wave potential (*E*_1/2_) determination was performed by CV scan in a 1.0 × 10^–3^ mmol L^–1^ [Fe­(CN)_6_]^3–/4–^ solution with 0.5 mol L^–1^ KCl over a potential interval from – 0.3 to 0.7 V (vs SCE) at a scan rate of 50 mV s^–1^. EIS measurements were carried out in the same solution over a frequency range from 75.000 to 20 Hz, with an applied potential of 0.22 V and a potential amplitude of 0.010 V (vs SCE). The applied potential was selected based on the *E*_1/2_ value obtained from CV. The same experiment was repeated using 0.50 mol L^–1^ KCl as a blank, with the same frequency range and half-wave amplitude, based on the CV results.

### 2.7 Differential Pulse Voltammetry (DPV) Measurements

DPV measurements were performed for Pb^2+^ and dopamine, both at a concentration of 50 μM. For Pb^2+^, voltammograms were recorded with and without a preconcentration step, which consisted of applying −1.1 V for 260 s under stirring prior to measurement. A 0.10 M KCl solution was used as the supporting electrolyte. The DPV parameters were: step potential, 2 mV; modulation amplitude, 50 mV; modulation time, 0.08 s; and scan rate, 25 mV s^–1^. These conditions were optimized based on previous reports in literature.^40^

For dopamine, measurements were conducted before and after surface activation, using 0.1 M PBS (pH 7.4) as the supporting electrolyte. The DPV parameters were: step potential, 2 mV; modulation amplitude, 25 mV; modulation time, 0.05 s; and scan rate, 100 mV s^–1^. These conditions were also optimized from literature.^41^

## 3 Results and Discussion

### 3.1 Physical and Morphological Characterization

Figure 2a,b presents a photograph of the polymer itself. The corresponding stress–strain curve is presented in Figure 2c. The ECO-MECO polymer exhibits a characteristic viscoelastic behavior, combining elastic and viscous deformation responses. At low strains (0–6.5%), the material displays a linear increase in stress with strain, indicating a predominantly elastic region in which deformation remains fully recoverable.^39^ Beyond this point, at the yield region, the polymer transitions to a plastic deformation regime, where strain continues to increase with little change in stress.

**Figure 2:**
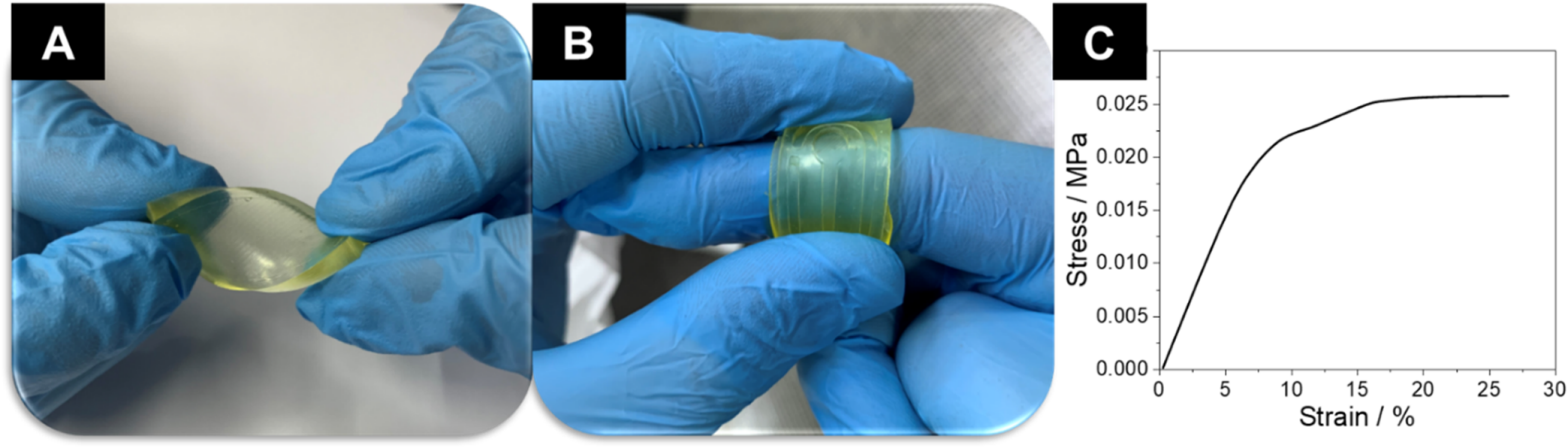
Photograph of the agglutinant ECO-MECO representing its flexibility that supports the possibility of use in wearable systems. (a) resistance to torsion; (b) resistance to folding of an imprinted electrode design and (c) stress–strain DMA curve.

TG/DTG curves (Figure 3) showed distinct mass losses when comparing the nonmodified polymer (ECO-MECO) with the corresponding composites G60EST40, G70EST30, and G80EST20, taking into account the same temperature interval. However, the decomposition of the polymer presented similar events in all curves, except by the burning of graphite in the composites. Description of the events and quantitative data on mass losses and their respective temperature ranges are presented in Table 2.

**Figure 3:**
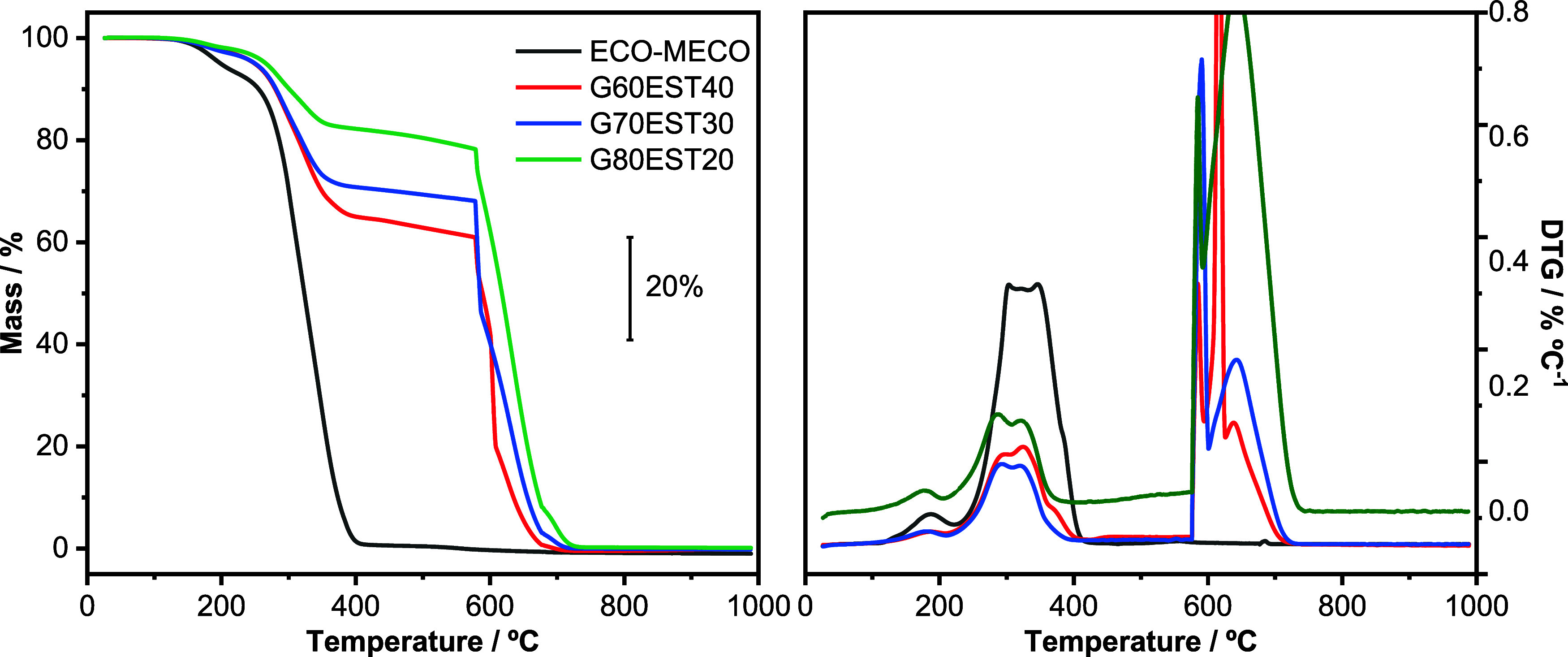
TG-DTG curves obtained for the polymer and the composites under a nitrogen atmosphere up to 600 °C (flow rate: 90 mL min^–1^), followed by a dynamic air atmosphere at the same flow rate. The analyses were carried out with a heating rate of 10 °C min^–1^, using an open platinum pan and approximately 6.0 mg of mass.

**Table 2:** Quantitative Data for Events, Mass Losses and Temperature Intervals for the Polymer and the Composites

sample	event	temperature Interval (°C)	mass loss (%)
ECO-MECO	volatiles release	127.8–223.7	6.77
	polymer degradation	223.7–533.4	91.0
	carbonaceous residue combustion	533.4–732.4	1.23
	residue	900	0.98
G60EST40	volatiles release	119.7–219.1	2.96
	polymer degradation	219.1–432.1	32.4
	carbonaceous residue pyrolysis	432.1–564.8	3.10
	graphite combustion	564.8–740.0	61.5
	residue	900	0
G70EST30	volatiles release	116.1–221.3	3.10
	polymer degradation	221.3–433.0	26.3
	carbonaceous residue pyrolysis	433.0–564.8	2.20
	graphite combustion	564.8–740.0	68.4
	residue	900	0
G80EST20	volatiles release	116.1–223.9	2.78
	polymer degradation	223.9–414.3	15.5
	carbonaceous residue pyrolysis	414.3–562.2	3.28
	graphite combustion	562.2–760.4	78.0
	residue	900	0

Under a nitrogen atmosphere, the ECO-MECO polymer exhibited thermal stability up to 122.8 °C, followed by three stages of mass loss. The first one related to volatiles as humidity, solvents and nonpolymerized monomers, followed by the polymer degradation and pyrolysis of carbonaceous material. A residue of *c.a.* 1% was observed at the end of the analysis, probably due to carbonaceous material (Table 2).

In the composite samples the decomposition of the polymeric fraction presented a similar profile, but with lower intensity in the decomposition steps. After the change in the atmosphere to air at 600 °C, polymer itself does not present any additional mass loss (black trace in Figure 2), while the composites presented an additional signal proportional to the graphite content in each case (red, blue and green traces in Figure 2). The graphite fractions determined after changing the atmosphere of furnace, were 61.5, 68.4 and 78.0%, respectively for G60EST40, G70EST30, G80EST20. The absence of residues at the end of TG curves of composites was attributed to the burning of graphite. These results confirmed the homogeneity of the samples and the expected carbon content in each one. No residues were found in the end of the TG experiments.

As the graphite content increases, the thermal stability of the composites is smaller: 280.0, 265.6, 262.1, and 254.5 °C, respectively for the unmodified polymer and for the composites G60EST40, G70EST30, G80EST20. The decrease in the initial temperature of decomposition may be related with the presence of agglomerates of polymer in the composites when compared to a bulk polymer sample (see SEM images below).

The absolute degradation rate also increased with higher content of graphite within the 282.5–334.4 temperature interval: Δ*m*/Δ*T* = −0.82, −0,28, −0.26 and −0.14% °C^–1^ (or −0.79; −0.72; −0.73; −0.70% °C^–1^, if the polymer content in the composite is considered) respectively for the unmodified polymer and for the composites G60EST40, G70EST30, G80EST20. The explanation for such difference is related to presence of graphite, which is not a good conductor of heat, promoting a retardation in the decomposition of the polymer.

SEM micrographs of fractured and polished surfaces, were obtained to better understand the morphological surface and bulk characteristics of the materials. Figure 4 presents the images of the polymer surface and fracture and of the composites, of both fractured and nonfractured polished areas.

**Figure 4:**
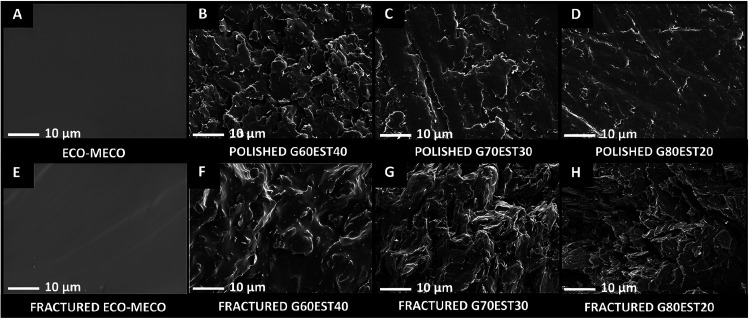
SEM micrographs at 2000× magnification. (a) ECO-MECO and polished surfaces of the samples: (b) G60EST40, (c) G70EST30, and (d) G80EST20. Fractured surfaces at the same magnification: (e) ECO-MECO, (f) G60EST40, (g) G70EST30, and (h) G80EST20.

Based on the SEM micrographs, the ECO-MECO polymer (Figure 4a,e) showed a smooth and homogeneous surface. When fractured, it maintains a similar morphology with few visible fracture lines, indicating internal cohesion of the material.

In the polished composites (G60EST40, G70EST30, and G80EST20), the surface roughness progressive decreased with the increment of graphite amount in the material. These images suggested that it is possible to have a polished smooth surface from 70 to 80% graphite wt %. The composite with 60 graphite wt %, remained rough even after polishing.

In the micrographs of the fractured samples, revealed the distribution of graphite within the internal volume of the composites. In the G60EST40 sample (Figure 4f), a greater presence of the polymer matrix surrounding the graphite particles is observed, suggesting better incorporation and possible adhesion between the phases. In the G70EST30 composite (Figure 4g) and G80EST20 (Figure 4h), the phases appear more distributed, with greater exposure of the graphite particles, a predominance of graphite planes is observed, with low binding by the polymer matrix, resulting in an irregular surface.

Wettability is an important feature of a composite candidate to be used as an electrode material. To better understand the interaction of water with the surfaces of the polymer and the polished composites, contact angle measurements were performed. The Figure 5 presents the images captured during the experiment.

**Figure 5:**
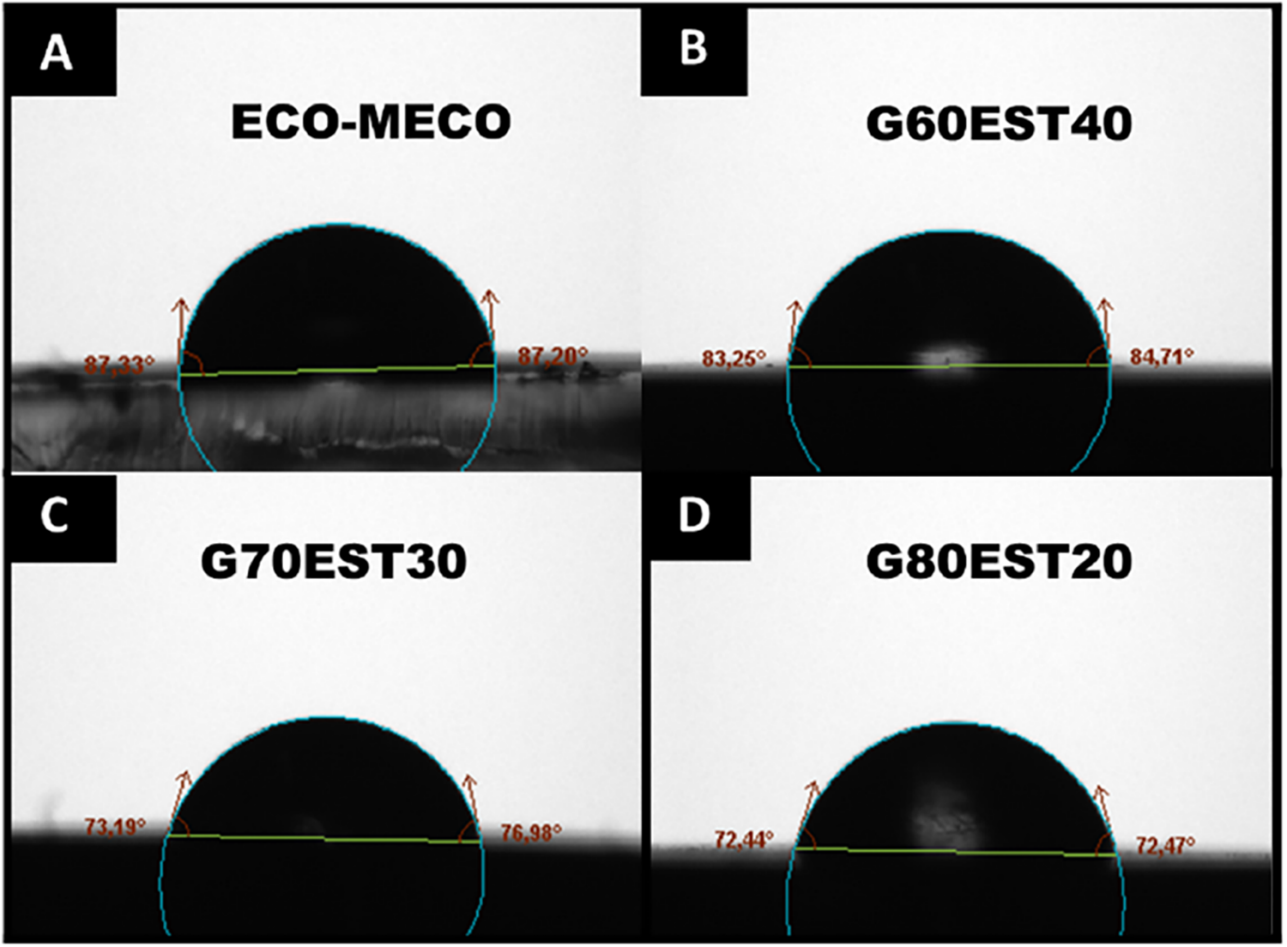
Contact angle measurements for the polymer (a) ECO-MECO (b) G60EST40, (c) G70EST30, and (d) G80EST20, respectively.

Based on Figure 5, a decrease in surface hydrophobicity is observed with the addition of graphite to the composite formulations, as evidenced by the reduction in contact angle: ECO-MECO (87.27 ± 0.39°), G60EST40 (83.98 ± 0.48°), G70EST30 (75.08 ± 1.78°) and G80EST20 (72.45 ± 3.54°). This behavior can be attributed to the greater affinity and adhesion of graphite to water molecules compared to the pure polymeric material. The presence of graphite enhances the wettability of the surface, consequently reducing its water-repellent capacity. Thus 70 and 80% (graphite, wt %) in composition seems to be the best formulations to use as a composite electrode.

### 3.2 Electrochemical Characterization of Electrodes

To evaluate the feasibility of using these composites in electroanalysis, cyclic voltammograms were obtained using 1.0 mmol L^–1^ [Fe­(CN)_6_]^3–/4–^ in 0.50 mol L^–1^ KCl at GCE and the composite electrodes G60EST40, G70EST30, and G80EST20, containing respectively 60, 70 and 80% (graphite, wt %). The resulting voltammograms are presented in Figure 6.

**Figure 6:**
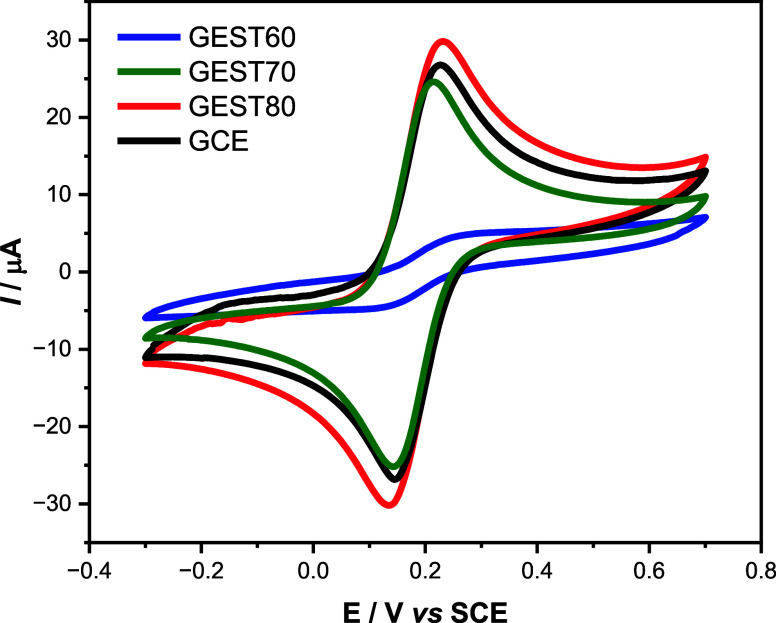
Cyclic voltammograms obtained for a 1.0 mmol L^–1^ [Fe­(CN)_6_]^3–/4–^ solution in 0.50 mol L^–1^ KCl, using a scan rate of 50 mV s^–1^, with electrodes containing different graphite proportions, compared to a glassy carbon electrode.

The 50:50 (wt %) composition was unsuitable for preparing solid composite electrodes, as the material lost its shape during the pressing step; however, it may still be applicable in other types of systems, like paste electrodes. In contrast, the 85:15 (wt %) composition exhibited a high graphite content relative to the polymeric binder, leading to poor cohesion, inhomogeneity, and compromised physical properties.

Voltammograms showed that the electrode containing 60% graphite did not exhibit a significant faradaic current response. Additionally, its voltammetric profile showed low surface activity, suggesting a limited electron transfer. This is in agreement with SEM images (Figure 4F) in which is clear that the polymer involves the graphite particles, what probably difficult the response of the conducting phase in the composite. Table 3 presents the parameters resulting from these voltammograms.

**Table 3:** Anodic and Cathodic Potential Values, Potential Variation, and Anodic and Cathodic Peak Currents Resulting from these Voltammograms

eectrodo	*E*_p,a_	*E*_p,c_	Δ*E*_p_	*I*_p,a_ / 10^–5^ ± SD^a^	*I*_p,c_ / 10^–5^ ± SD^a^	RSD (%)*I*_p,a_ – *I*_p,c_
GCE	0.226	0.146	0.080	2.5 ± 0.1	–2.52 ± 0.03	4.0–1.1
G70EST30	0.215	0.145	0.070	2.5 ± 0.4	–2.6 ± 0.3	16.0–12.5
G80EST20	0.232	0.136	0.096	2.8 ± 0.9_7_	–2.9 ± 0.5	32.1–17.2

a Mean ± standard deviation, *n* = 3.

However, the voltammetric response at G70EST30 and G80EST20 was well-defined and with peak currents comparable of that from GCE in shape and intensity. Quantitative data regarding these experiments are presented in Table 3.

From this table is possible to observe that both composite electrodes even without any surface treatment, presented similar responses to that of GCE in profile. Peak currents and potentials at G70EST30 and GCE are close, while at G80EST20 presented a peak current is 12% higher than at GCE, however it presented some crumbling during polishment. This reveals that both composites are promising as electrode materials.

Thus, considering the electrochemical responses, mechanical features and easiness of fabrication, the G70EST30 electrode was selected for further studies.

### 3.3 Surface Treatment of Composite Electrode

To enhance the electrochemical performance of the electrode, particularly in terms of peak current responses and surface activity, the G70EST30 was subjected to an electrochemical activation. During this treatment, two commonly used inner and outer-sphere redox probes were used: the anionic probe [Fe­(CN)_6_]^3–/4–^ and the cationic probe [Ru­(NH_3_)_6_]^3+/2+^, respectively, both at a concentration of 1.0 mmol L^–1^ in 0.50 mol L^–1^ KCl solution. Cyclic voltammograms were recorded at every 50 activation cycles within the potential window of – 1.0 to +1.5 V (vs Ag/AgCl) in 0.10 mol L^–1^ phosphate solutions at pH 3.0, 4.0, and 7.0, using a scan rate of 200 mV s^–1^.

There are a variety of surface activation procedures, the most usual: involves thermal, photo and voltammetric activation. Among the voltammetric ones, cycling in phosphate buffer is widely known and a usual choice.^42,43^ The primary objective of this electrochemical treatment was to promote the formation of oxygen-containing functional groups on the graphite surface, thus increasing its electroactive area and facilitating charge transfer, as evidenced by enhanced peak currents.^42,43^

Representative cyclic voltammograms, along with the corresponding anodic and cathodic peak currents obtained during the electrochemical treatment, are presented in Figures S1 (pH 3.0), S2 (pH 4.0), and S3 (pH 7.0) of the Supporting Information. The mean peak current values determined under these conditions (n = 3) are summarized in the Figure 7.

**Figure 7:**
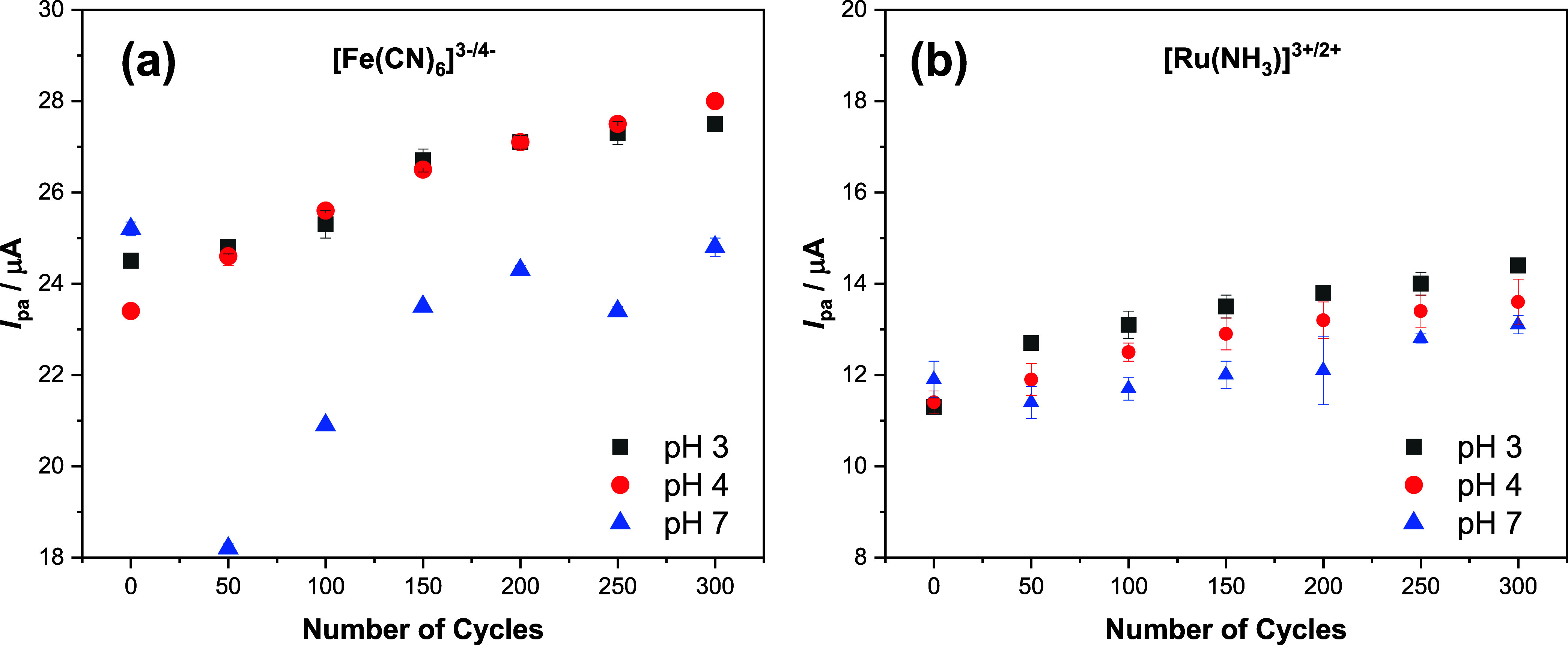
Comparison of the electrochemical treatments performed in phosphate solutions at pH 3.0, 4.0, and 7.0. The results were obtained using (a) 1.0 mmol L^–1^ [Fe­(CN)_6_]^3–/4–^ in 0.50 mol L^–1^ KCl solution and (b) 1.0 mmol L^–1^ [Ru­(NH_3_)_6_]^3+/2+^ in 0.50 mol L^–1^ KCl solution.

It was observed that, prior to electrochemical treatment, the peak currents were of similar magnitude in the different pH for each redox probes. After 50 activation cycles, a slight increase in peak current intensity was noted at pH 3.0 and 4.0, which continued with further cycling. This increase reached a plateau after approximately 150–200 cycles under both conditions.

At pH 7.0, however, the peak current for the [Fe­(CN)_6_]^3–/4–^ probe sharply decreased after 50 cycles followed by a gradual increase with subsequent treatment cycles. The peak current stabilization occurred after 200 cycles at a lower magnitude than that the one previously measured for the untreated electrode. A similar behavior was observed for the ruthenium complex, with less variation in current intensity due to the lower reactivity of this probe. According to literature report,^44^ although the Ru­(NH_3_)_6_^3+/2+^ redox couple is considered electrochemically reversible, it exhibits slower heterogeneous electron transfer kinetics on graphite based electrodes compared to the ferro/ferricyanide.

Before electrochemical treatment, regardless of pH, the electrode surface can be assumed to be relatively polished, exposing a fraction of the condutive phase. This condition accounts for the initial electrochemical response prior to cycling.

The behavior observed at pH 7.0 during treatment can be attributed to two primary factors. The first involves the binder phase: after 50 cycles, the activation of functional groups within the polymer matrix may hinder charge transfer reactions. As previously discussed, this effect is more pronounced for the more reactive ferrocyanide couple than for the ruthenium complex. Infrared analysis of the binder (Figure S4) revealed characteristic absorption bands (in cm^–1^) associated with −OH deformation at ∼3500, CH_2_ symmetric and asymmetric stretching at 2926 and 2856, −C=O stretching at 1734, ester groups (R_1_C­(O)­OR_2_) at 1155, and C=C stretching at 998. These functionalities are consistent with the polyester structure used as a binder. However, under electrochemical treatment, they may undergo redox processes within conductive domains, as well as acid–base reactions, especially at elevated pH, involving pendant carboxylic groups in the polymer matrix.

The second factor involves the progressive activation of the conductive phase during electrochemical cycling in phosphate buffer. The main objective of the electrochemical treatment is to activate the electrode surface, thus enhancing peak currents and facilitating charge transfer. With increasing scan cycles, phosphate anions interact with the electrode surface, leading to the formation of functional groups such as hydroxy, carbonyl, and carboxy, which is typical products of graphite oxidation.^43^ This process progressively activates defect sites within the graphitic structure, thus improving electron transfer kinetics. This structure behavior its similar to defects of graphene oxide^45^

The improved electron transfer observed in subsequent cycles can be attributed to this surface activation, as the functional groups within the binder are primarily affected during the initial 50 cycles. Therefore, further current gains result predominantly from the graphite modification.

At pH 3.0 and 4.0, the functional groups are expected to be protonated and consequently, less active. Under these conditions, the current increase observed for both probes are solely attributed to the activation of the graphite phase.

A similar influence of pH and electrochemical treatment was observed on cathodic peak currents. Table 4 presents the peak current values obtained after 150 activation cycles in comparison to those from untreated electrodes. Based on the results of this study, it can be concluded that electrochemical treatment in phosphate buffer is effective at pH 3.0 and 4.0, leading to significant current enhancement up to at least 150 cycles. However, at pH 7.0, no substantial gain in current was observed that would justify the use of this treatment.

**Table 4:** Initial Anodic Peak Current, Anodic Peak Current after 150 Cycles in Cyclic Voltammetry, and the Corresponding Current Variation for [Fe­(CN)_6_]^3–/4–^ and [Ru­(NH_3_)_6_]^3+/2+^

	anodic current (μA)					
	[Fe(CN)_6_]^3–/4–^			[Ru(NH_3_)]^3+/2+^		
pH	initial	after 150 cycles	change (%)	initial	after 150 cycles	change (%)
3.0	24.5	26.7	9.0	11.3	13.5	19.5
4.0	23.4	26.5	13.2	11.4	12.9	13.2
7.0	25.2	23.5	–6.7	11.9	12	0.8

Repetability and reproducibility of the G70EST30 composite electrode were also evaluated by cyclic voltammetry of [Fe­(CN)_6_]^3–/4–^ 1.0 mmol L^–1^, in KCl 0.50 mol L^–1^, in three sets of 6 scans, with surface renovation between each set. The results are presented in Table 5.

**Table 5:** Repeatability (Intraday) and Reproducibility Studies on the G70EST30 Composite Electrode, Using [Fe­(CN)_6_]^3–/4–^ 1.0 mmol L^–1^, in KCl 0.50 mol L^–1^, Cyclic Voltammetry, in Three Sets of 6 Scans, with Surface Renovation between Each Set

	results^a^			
experiment set	*E*_p,a_ (V)^b^	*I*_p,a_ (μA)	*E*_p,c_ (V)^b^	*I*_p,c_ (μA)
1	0.102 ± 0.003	–21.7 ± 0.0_0_	0.282 ± 0.003	21.6 ± 0.0_0_
2	0.117 ± 0.002	–20.9 ± 0.0_0_	0.271 ± 0.004	20.4 ± 0.0_0_
3	0.122 ± 0.002	–20.0 ± 0.0_0_	0.266 ± 0.001	19.4 ± 0.0_0_

a mean ± standard deviation, *n* = 6.

b vs. SCE.

The results in each experiment set in Table 5 represent the repeatability of the electrode response between six successive measurements. The reproducibility is represented by the responses in the columns of this table, after surface renovation, as described in the Section 2.

It was also possible to claim that the electrode is stable for at least 5 months, once a single device was used during this time to obtain the measurements described in this work, it was not possible to evaluate the interdays reproducibility once the electrode surface was renewed in each working day.

### 3.4 Electrochemical Impedance Spectroscopy (EIS)

EIS measurements were performed for the G30EST70 electrode in a solution [Fe­(CN)_6_]^3–/4–^, using electrodes with and without prior electrochemical treatment in phosphate solution (pH 4) for up to 300 cycles. This pH was chosen once the previous results showed a higher peak of current gain. The results were compared with those obtained for an untreated GCE. Figure 8 presents the corresponding Nyquist and Bode plots.

**Figure 8:**
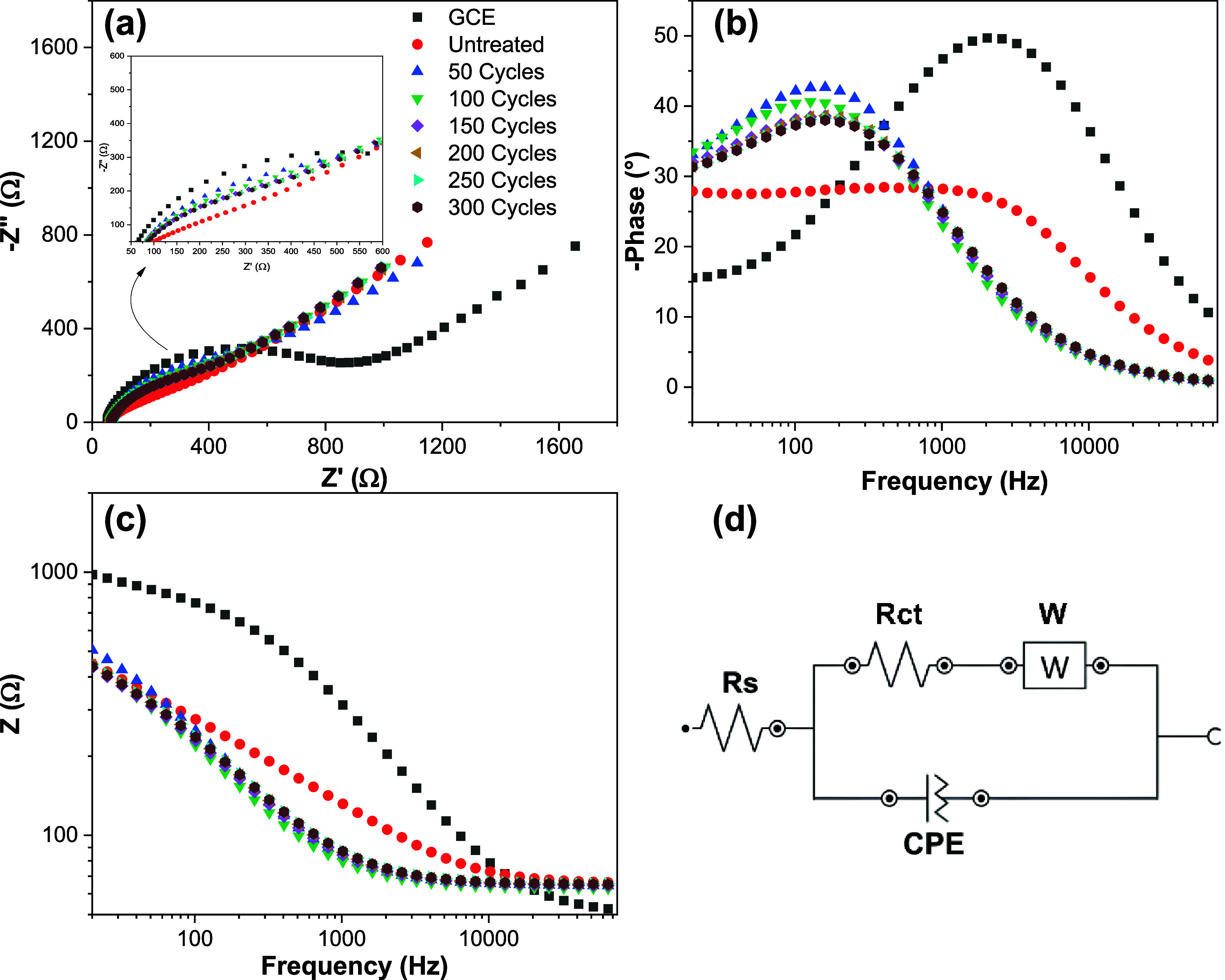
EIS data obtained in 1.0 mmol L^–1^ [Fe­(CN)_6_]^3–/4–^ with 0.50 mol L^–1^ KCl: (a) Nyquist plot; (b) Bode plot–phase angle vs frequency; (c) Bode plot–impedance modulus (|Z|) vs frequency; (d) corresponding equivalent electrical circuit model.

In the Nyquist plot (Figure 8a), the untreated GCE showed a well-defined semicircle, characteristic of a charge transfer process governed by polarization resistance and double-layer capacitance. In contrast, the untreated G30EST70 electrode does not exhibit a similarly defined semicircular profile. However, after electrochemical treatment in phosphate solution and with increasing cycle number, the formation of a progressively more semicircular pattern is observed. Despite this evolution, the profile remains distinct from that of the GCE, likely due to the higher capacitance and surface heterogeneity of the modified material. To proper model surface heterogeneity regarding electric double layer charge and discharge, constant phase element (CPE) was the electrical circuit element of choice to better represent the capacitive behavior of the electrode interface. This is evidenced by the α values below 1 associated with an ideal capacitor which is a consequence of a homogeneous and flat surface.

In the Bode plots (Figure 8b,c), a shift in the frequency corresponding to the maximum phase angle is observed as the electrodes undergo electrochemical treatment. The glassy carbon electrode exhibits a phase behavior similar to that of the untreated G30EST70 electrode, although with a greater variation in phase angle. The observed shift toward lower logarithmic frequencies suggests a transition from predominantly resistive to more capacitive behavior.^46^ This phenomenon can be attributed to the charging and discharging of the electrical double layer induced by potential oscillations during the sinusoidal perturbation.^47^ In this transient regime, the electrolyte solution resistance (*R*_s_) initially exerts the greatest influence. As the frequency decreases, the contribution of solution resistance and ohmic drop becomes less significant, and the impedance associated with the capacitive component of the system, namely, the double-layer capacitance, becomes dominant. This occurs because the double-layer charging process responds more rapidly than resistive and charge transfer processes.^47^

The shift in the frequency associated with the phase angle maximum may also be related to the density of functional groups on the binder electrode surface (Figure S5). Both the GCE and the untreated G30EST70 electrodes possess relatively few surface functional groups capable of accumulating electrical charge, which limits the capacitive behavior of the system. In contrast, after electrochemical treatment in phosphate solution and with increasing cycle number, a significant surface modification occurs, likely due to the formation and growth of carboxylate functional groups.^48,49^ This is evidenced by the increase in admittance values (*Y*_0_) nearly 4-fold the untreated surface which are directly related to interfacial capacitance with the increasing number of cycles.

These functional groups facilitate charge accumulation at the electrode/solution interface, promoting higher capacitive behavior, particularly at low frequencies. As a result, the treated electrode begins to exhibit characteristics typical of an electrochemical capacitor, which explains the shift observed in the Bode plots.

Furthermore, an increase in the number of treatment cycles leads to a slight reduction in the phase angle variation at a given logarithmic frequency, suggesting enhanced stabilization of the electrode/solution interface and improved surface homogeneity of the treated electrode.

Assuming that all charge-transfer elements in the electrochemical system can be represented by physical components within an equivalent electrical circuit, and based on the Nyquist plot results (Figure 8a), an equivalent circuit model was proposed (Figure 8d). The corresponding fitting results obtained from this model are summarized in Table 6.

**Table 6:** Simulated Parameters Obtained from the Equivalent Circuits

0.50 mol L^–1^ KCl							
	number of treatment cycles						
fitted parameters	0	50	100	150	200	250	300
*R*_s_/Ω	76.2	70.4	73.0	67.5	70.9	70.7	70.4
*Y*_0_/μMho*s^(N)^	3.22	9.4	11.2	9.98	9.56	9.49	9.4
α	1	0.92	0.92	0.91	9.91	0.91	0.91
χ2 (0.10^–3^)	0	22	15	23	24	26	26
	0.50 mol L^–1^ KCl + 1.0 mmol L^–1^ K_3_[Fe(CN)_6_]^3–/4–^						
*R*_s_/Ω	59.2	63.6	62.2	63.5	64.1	64.6	64.1
*R*_ct_/Ω	403	493	408	373	383	373	368
*Y*_0_ (μMho*s^(N)^)	36.6	18.6	22.5	21.8	20.3	20.4	21.4
α_1_ (CPE_1_)	0.62	0.83	0.82	0.81	0.81	0.81	0.80
*W* (μMho*s^(N)^)	314	416	424	419	425	417	419
χ^2^ (0.10^–4^)	41	43	25	45	45	44	46

As shown in the equivalent circuit (Figure 8d), Rs corresponds to the electrolyte solution resistance, Rct represents the charge transfer resistance at the graphite–electrode surface interface and at the electrode/probe interface, and the CPE (constant phase element) accounts for the nonideal capacitive behavior of the system

To determine the most appropriate arrangement of elements in the equivalent circuit, the fit quality was evaluated based on the chi-squared statistic (χ^2^), in addition to a physicochemical interpretation of each circuit component, as detailed in Table 6.

Subsequently, the same experiment was performed in a KCl solution without the redox probe to evaluate the electrode surface response in the absence of electroactive species. Figure S5 presents the corresponding electrochemical impedance spectra and the proposed equivalent circuit.

According to the Nyquist plot (Figure S5a), in KCl solution, differences in the slope of the real and imaginary components indicate evidence of internal charge transfer processes on the electrode surface, for both untreated and treated electrodes.

In the Bode plots (Figure S5b,c), analysis of the phase angle also reveals the presence of a resistive and capacitive element, similar to that observed in Figure 8. Initially, the system is governed by the solution resistance, which is responsible for charging the electrical double layer, thus generating system reactance. In contrast, in the ferrocyanide-containing solution (Figure 8), the system exhibits typical pseudocapacitive behavior, as indicated by the earlier onset of the phase angle peak. This phenomenon occurs due to the presence of a redox-active species, which allows charge flow beyond the mere formation of the electrical double layer. This implies rapid faradaic processes and ion movement at the electrode/solution interface, enabling more efficient charge storage. Such behavior does not occur in KCl-only solution, where no redox-active species are present. In this case, the main contribution to the electrical response arises from the double-layer capacitance, resulting in phase angles approaching 90°, indicative of nonideal capacitive behavior.

Comparison of the impedance modulus as a function of frequency (Figure S5c) shows that the untreated G30EST70 electrode deviates from the response of the glassy carbon electrode, and this deviation becomes more pronounced following electrochemical treatment. This trend supports the hypothesis that the treatment increases the electrode’s capacitance. The charge transfer elements involved in the electrolyte response were modeled using an equivalent circuit (Figure S5d), with the fitting parameters summarized in Table 6.

The electrochemically active surface area (ECSA) was evaluated using the Randles–Ševčík Equation^50,51^ (eq 1) and the results from voltammograms of 1.0 mmol L^–1^ [Fe­(CN)_6_]^3–/4–^ redox probe in 0.5 mol L^–1^ KCl, over a scan rate range of 5.0–200 mV·s^–1^, at the G70EST30 electrode (Figure 9a) and GCE (Figure 9b).

1 $$I_{p}=\left(2.69\times 10^{5}\right)n^{3/2}AD^{1/2}{\mathrm{Cv}}^{1/2}$$

In which *I*_p_ is the peak current, *n* is the number of electrons involved in the redox process, *A* is the electrochemically active surface area (cm^2^), *D* is the diffusion coefficient of the electroactive species (7 × 10^–6^ cm^2^·s^–1^), *C* is its bulk concentration (mol·cm^–3^), and ν is the scan rate (V·s^–1^).

**Figure 9:**
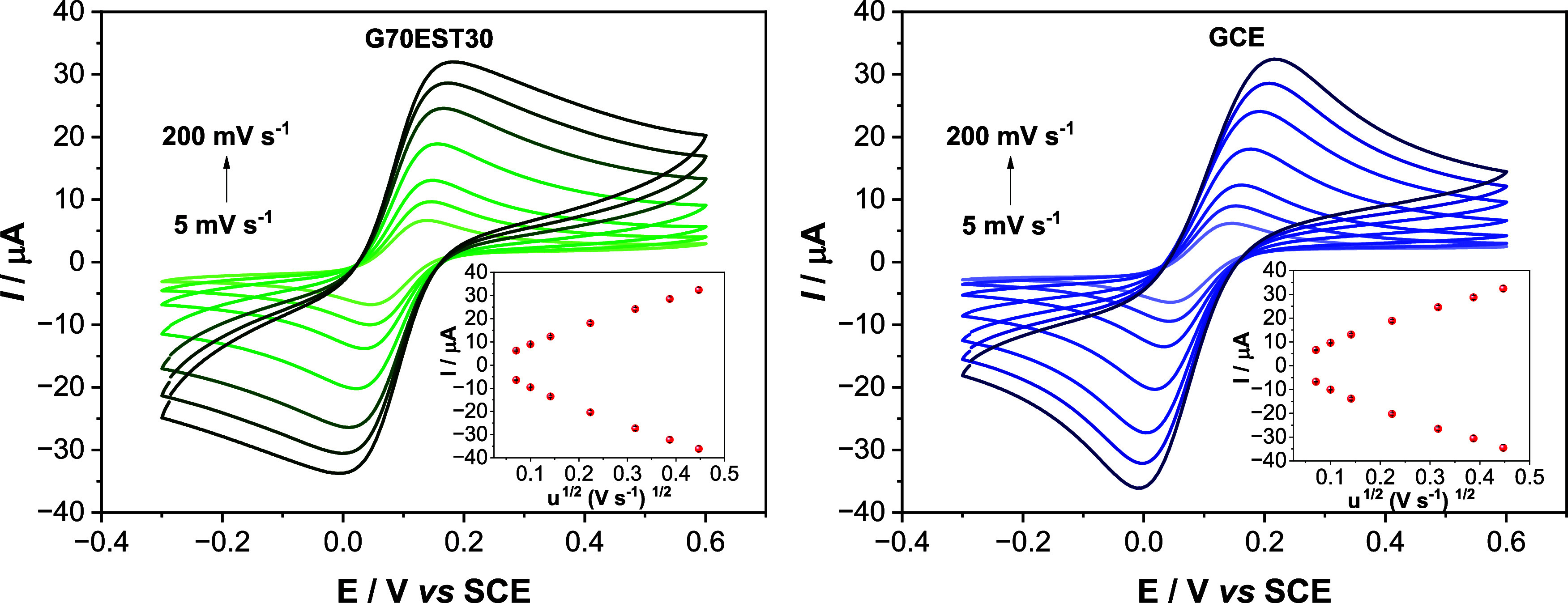
Cyclic voltammetry responses at different scan rates (5.0–200 mV s^–1^) using 1.0 mmol L^–1^ [Fe­(CN)_6_]^4‑/3‑^ redox probe in 0.50 mol L^–1^ KCl for (a) G70EST30 and (b) GCE. Insets: Randles–Sevcik plots.

Thus, plot of *I*_p_ vs ν^1/2^ was draw and the active area was calculated from the slope of the resulting straight lines. The active areas are presented in Table 7.

**Table 7:** Electroactive Area, *R*_ct_, and Heterogeneous Electron Transfer Constants (*k* ^0^) of the GCE and G70EST30 Electrodes^a^

electrode	electroactive area^b^ (cm^2^)	*R*_ct_ ^c^ (Ω)	*k* ^*0*^ (cm·s^–1^)
GCE	0.096 ± 0.005	797	(3.5 ± 0.2) × 10^–3^
G70EST30	0.097 ± 0.003	373	(7.4 ± 0.2) × 10^–3^

a Geometric área: 0.0707 cm^2^ (ϕ = 3.0 mm).

b Mean ± standard deviation, *n* = 3.

c Values obtained by EIS (Table 6).

Prior to measurements, the G70EST30 surface was electrochemically pretreated in a phosphate solution at pH 4, as described above. The ECSA values obtained were 0.097 ± 0.003 cm^2^ for G70EST30 and 0.096 ± 0.003 cm^2^ for the GCE. These comparable values indicate that the G70EST30 electrode exhibits an active surface area similar to that of the GCE.

Subsequently, the heterogeneous electron transfer constants (*k* ^0^) for both electrodes were calculated using eq 2.^52^

2 $$R_{\mathrm{ct}}=\frac{\mathrm{RT}}{k^{0}n^{2}F^{2}\mathrm{AC}}$$

in which *R*_ct_ is charge transfer resistance, *R* is the gas constant (8.314 J mol^–1^ K^–1^), *F* is the Faraday’s Constant (96485 C mol^–1^), *T* is the absolute temperature, *n* is the number of electrons involved in the redox process, *A* is the electrochemically active surface area (cm^2^), *C* is its bulk concentration (mol·cm^–3^), and κ^0^ is the heterogeneous electron transfer rate (cm·s^–1^).

Despite their similar electroactive surface areas, the electrodes exhibited different heterogeneous electron transfer constants, with G70EST30 at (7.44 ± 0.23) × 10^–3^ cm·s^–1^ and GCE at (3.46 ± 0.17) × 10^–3^ cm·s^–1^. Thus, the G70EST30 electrode provides more than twice the electron transfer rate compared to the GCE (Table 7).

### 3.5 Analyte Potentialities Evaluation

To evaluate the applicability of the proposed electrode, its electrochemical response for Pb^2+^ an inorganic specie and dopamine as an organic compound was evaluated. Lead was selected as an environmental contaminant, while dopamine represents a biologically relevant neurotransmitter.

The performance of the G70EST30 electrode was examined before and after surface activation for dopamine detection. In the case of Pb^2+^ the performance was evaluated without electrochemical pretreatment but before and after preconcentration step. These experiments were designed to explore the electrode surface response under different analytes and analytical strategies. The parameters optimization were based on literature using electrodes with composition using graphite derivatives.^40,41^ The results obtained for Pb^2+^ and dopamine are presented in Figure 10.

**Figure 10:**
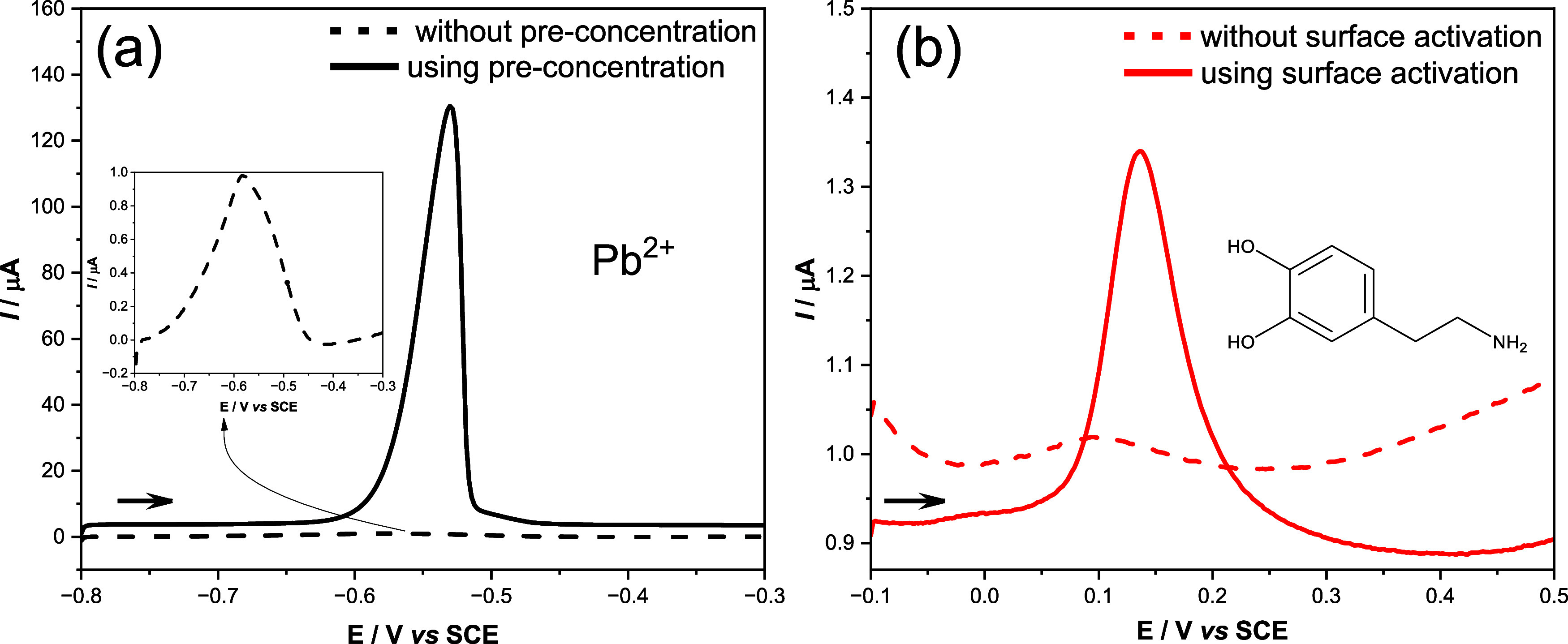
Differential pulse voltammograms of (a) Pb^2+^ recorded without and with a preconcentration step (50 μM, 0.1 M KCl) and (b) dopamine (50 μM, 0.1 M PBS, pH 7.3) obtained before and after surface activation. Experimental parameters: step potential, 2 mV; modulation amplitude, 25 mV; modulation time, 0.05 s; scan rate, 25 mV s^–1^. For Pb^2+^, a conditioning step at −1.1 V for 260 s under stirring was performed prior to measurement.

The voltammograms in Figure 10a exhibited a huge improvement in the response for Pb^2+^ after the preconcentration step, even without surface treatment. This can be related to the action of organic groups in the polymer that can interact with the ionic species facilitating its accumulation at the electrode surface.

Figure 10b presents the remarkable effect of the surface activation in the response of the organic analyte when compared to the untreated surface, reveling the importance of the functional groups generated on the electrode surface in this case.

These findings indicate that the chemical functional groups of the binder can facilitate metal deposition on the electrode surface during the preconcentration process, while surface treatment enhances the detection of organic compounds such as dopamine.

## 4 Conclusion

This work demonstrated the possibility of obtaining a new flexible polymeric material from castor oil, a renewable and sustainable resource, after epoxidation and maleinization. This new material was used in the preparation of solid composite electrodes as a nonconductive binder, using graphite as the conductive phase. Thermogravimetric studies showed a homogeneous distribution of graphite within the composite, which was corroborated by microscopy images.

The electrochemical surface treatment, performed by potential cycling in a phosphate medium, was evaluated to elucidate the electrode response and to assess the activation effects of both the graphite and the functional groups present in the binder, employing two electrochemical probes with distinct physicochemical properties.

The potentialities of the material for analytical applications toward both organic and inorganic analytes was demonstrated. Notably, the functional groups of the binder enabled the preconcentration of Pb^2+^ as a representative example of metal ion analysis, without the need for additional surface modifiers. Following electrochemical activation, a marked enhancement in the electrochemical response toward dopamine was observed, highlighting the suitability of this novel electrode material for sensing applications.

The high flexibility of the polymer suggests its potential use in the fabrication of flexible electrodes, including wearable sensing systems. In addition, the results obtained indicate a wide range of prospective analytical applications for this material in future developments.

## Supplementary Material



## Notes

Voltammograms obtained during the electrochemical surface activation at different pH values in phosphate buffer solution, FTIR spectra of the polymer, and the Nyquist and Bode plots in KCl (PDF)
