# Supplementary material for: Composites Electrodes Based on Castor Oil Derivatives and Graphite: Synthesis, Properties and Electroanalytical Applications
Source: ACS Omega. 2025 Dec 10;10(50):62243–56. doi: 10.1021/acsomega.5c10053 (PMC12750197; doi:10.1021/acsomega.5c10053)
Supplement: Supplementary file 1 [file ao5c10053_si_001.pdf]

Composites Electrodes Based on Castor Oil Derivatives and Graphite: Synthesis, Properties and Electroanalytical Applications

Jonatha de Freitas, Rafael da Silva, Rafael Martos Buoro, Rafael Turra Alarcon, Éder Tadeu Gomes Cavalheiro

Universidade de São Paulo-USP, Instituto de Química de São Carlos, 13566-590, São Carlos, SP, Brazil.

Supporting Information

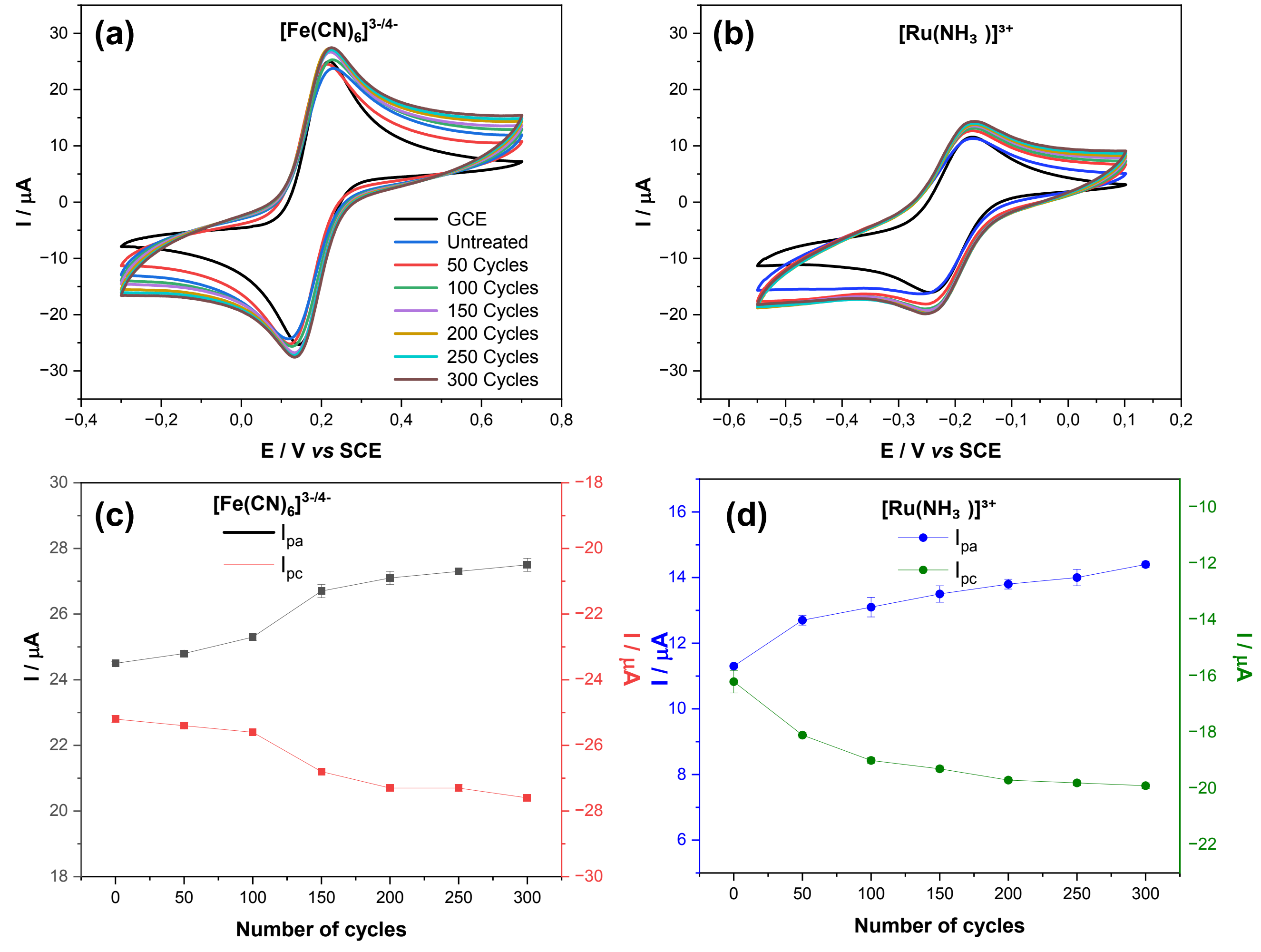

Figure S1. Cyclic voltammograms obtained from the electrode treated in pH 3 phosphate solution for (a) 1.0 mmol L⁻¹ $K_3[Fe(CN)_6]$ in 0.50 mol L⁻¹ KCl and (b) 1.0 mmol L⁻¹ $[Ru(NH_3)_6]Cl_3$ The corresponding anodic and cathodic peak currents are shown in (c) for $K_3[Fe(CN)_6]$ and in (d) for $[Ru(NH_3)_6]Cl_3$. Scan rate of 50 mV s⁻¹ was used with the G70EST30 electrode, at different scan cycles, within a potential range of -1.0 V to 1.5 V.

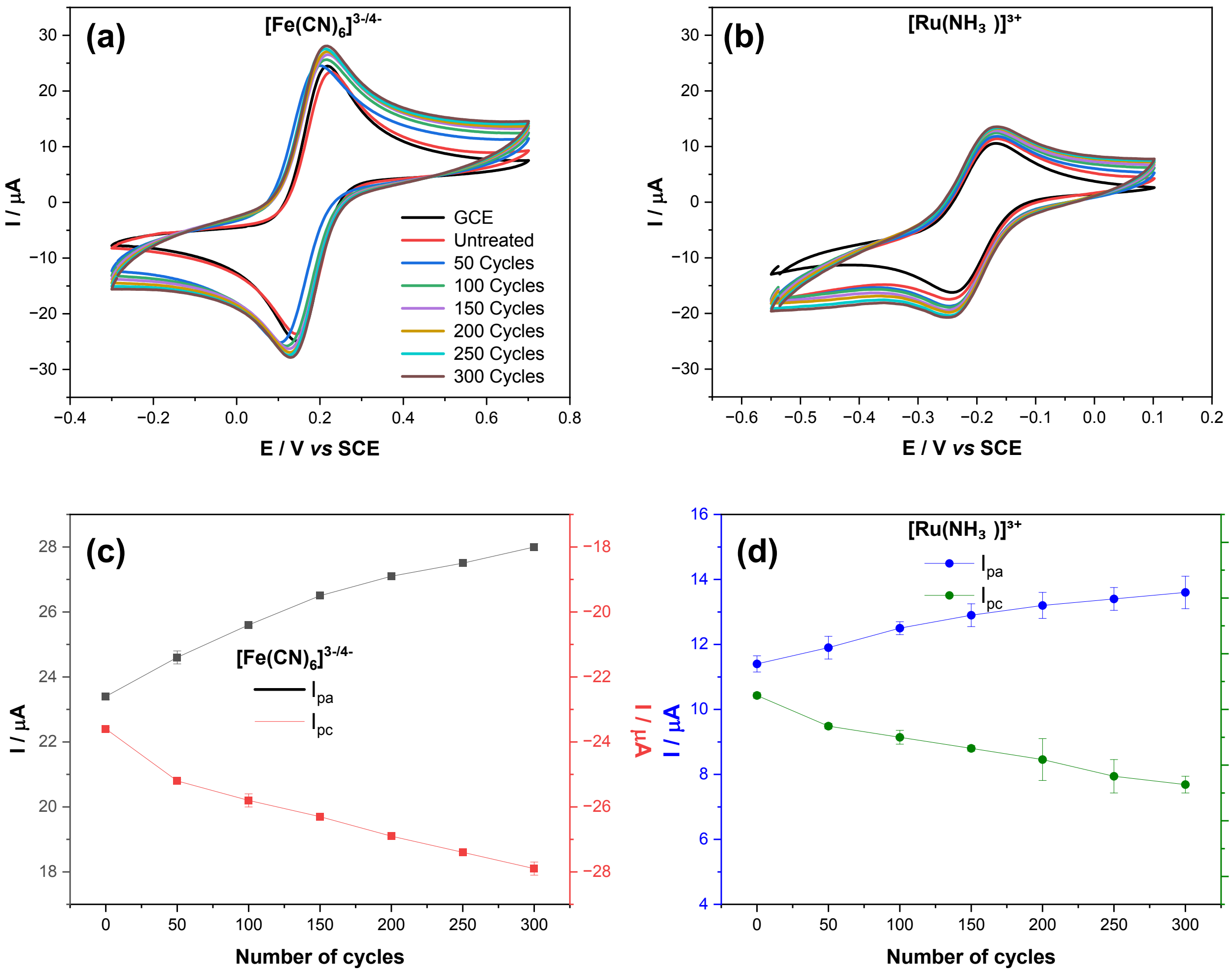

Figure S2. Cyclic voltammograms obtained from the electrode treated in pH 4 phosphate solution for (a) 1.0 mmol L^{-1} $K_3[Fe(CN)_6]$ in 0.50 mol L^{-1} KCl and (b) 1.0 mmol L^{-1} $[Ru(NH_3)_6]Cl_3$ The corresponding anodic and cathodic peak currents are shown in (c) for $K_3[Fe(CN)_6]$ and in (d) for $[Ru(NH_3)_6]Cl_3$. Scan rate of 50 mV s^{-1} was used with the G70EST30 electrode, at different scan cycles, within a potential range of -1.0 V to 1.5 V.

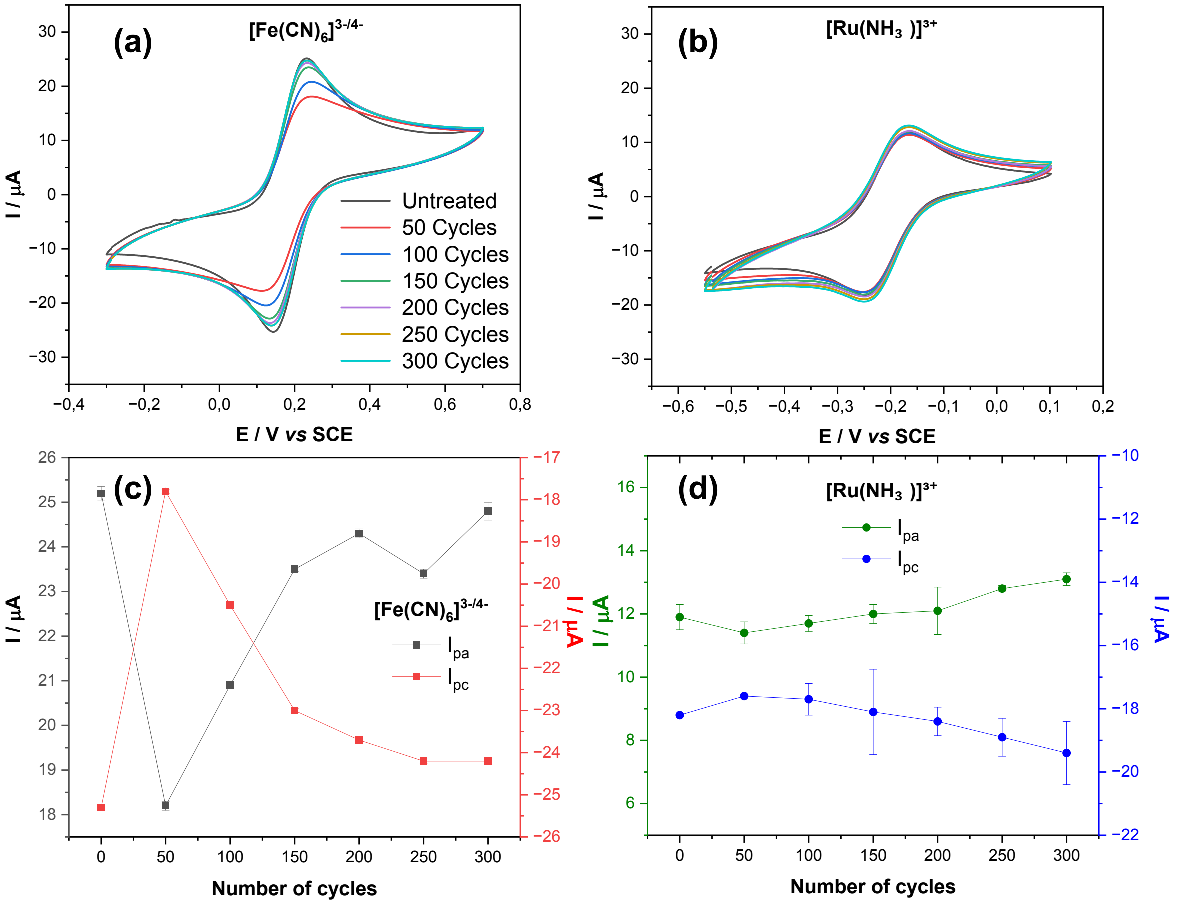

Figure S3. Cyclic voltammograms obtained from the electrode treated in pH 7 phosphate solution for (a) 1.0 mmol L^{-1} $K_3[Fe(CN)_6]$ in 0.50 mol L^{-1} KCl and (b) 1.0 mmol L^{-1} $[Ru(NH_3)_6]Cl_3$ The corresponding anodic and cathodic peak currents are shown in (c) for $K_3[Fe(CN)_6]$ and in (d) for $[Ru(NH_3)_6]Cl_3$ Scan rate of 50 mV s^{-1} was used with the G70EST30 electrode, at different scan cycles, within a potential range of -1.0 V to 1.5 V.

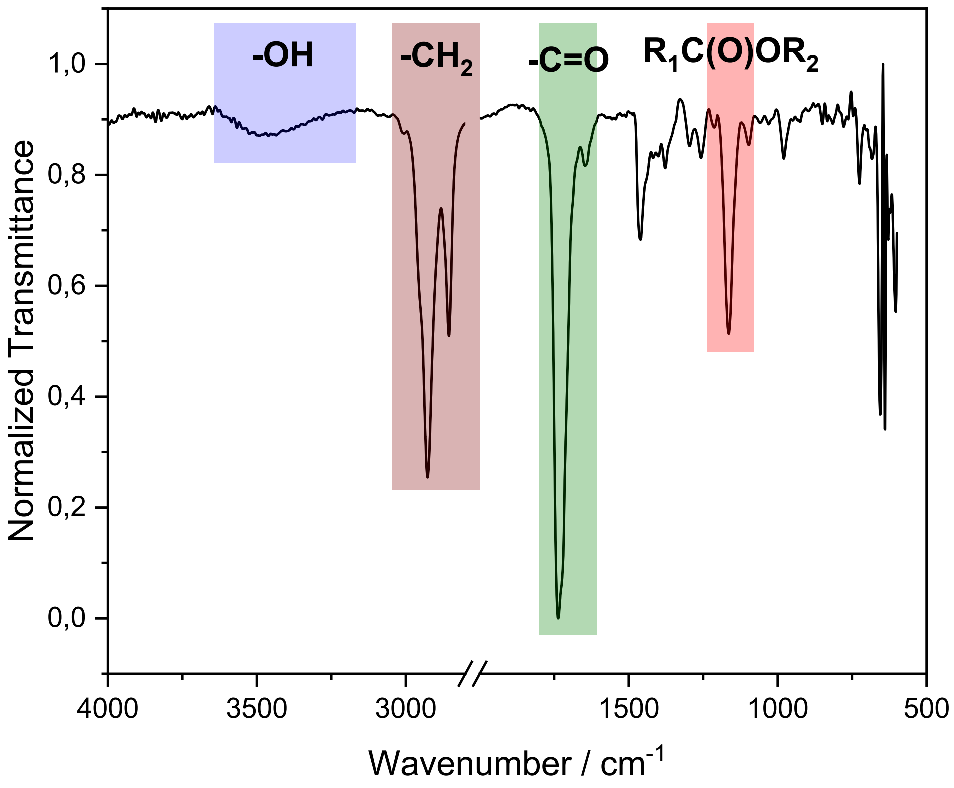

Figure S4. MIR spectra for polymer.

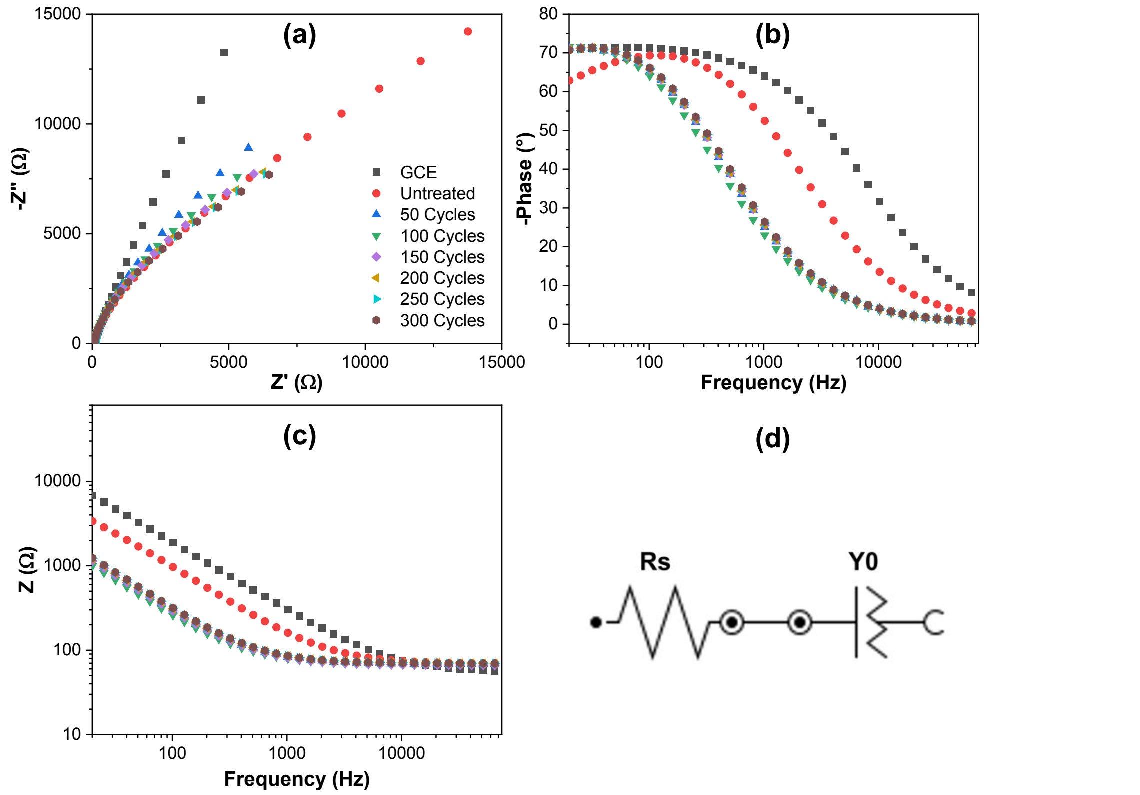

Figure S5. EIS data obtained in 0.50 mol L⁻¹ KCl: (a) Nyquist plot; (b) Bode plot – phase angle vs. frequency; (c) Bode plot – impedance modulus (|Z|) vs. frequency; (d) corresponding equivalent electrical circuit model.
